# Interaction of Pf4 tactoids with bacteria and synthetic colloidal rods

**DOI:** 10.1007/s00249-026-01844-5

**Published:** 2026-05-11

**Authors:** Mariana de Oliveira Silva, Iago Grobas, Abul K. Tarafder, Tanmay A. M. Bharat, Dirk G. A. L. Aarts

**Affiliations:** 1 Department of Chemistry, Physical and Theoretical Chemistry Laboratory, University of Oxford, Oxford, UK; 2 Theoretical and Computational Systems Biology Program, Institute for Integrative Systems Biology (I2SysBio), CSIC-UV, Valencia, Spain; 3 Structural Studies Division, MRC Laboratory of Molecular Biology, Cambridge, UK

## Abstract

We investigated the interaction of liquid crystal droplets, formed by a phase separating system of elongated virus particles and polymer with rod-like impurities. The virus particles were Pf4, which when mixed with sodium alginate as polymer, phase separated into an isotropic phase in coexistence with a nematic phase of the virus particles. The nematic phase appeared in the form of spindle-shaped liquid crystal droplets, which are called tactoids. We studied the interaction of this mixture with micrometer sized rods, which were either bacteria (*Bacillus subtilis* or *Escherichia coli*) or inert colloidal particles (made of SU8 photoresist polymer or silica). Confocal laser scanning microscopy was used to examine the mixtures and four different dominant configurations were found, classified as no attachment, partial attachment of the rod to the tactoid, a “sandwich” state, where two tactoids interact with the same rod, and lastly encapsulation of the rods by the tactoids. We categorized the results in terms of surface properties and rod geometry (size and aspect ratio). We further investigated the system through numerical calculation in a simplified two-dimensional model. Finally, we addressed the role of confining the experimental system from three dimensions to a quasi two-dimensional setup, a relevant scenario in bacteria biofilms.

## Introduction

*Pseudomonas aeruginosa* is a major human bacterial pathogen responsible for significant morbidity and mortality among patients with cystic fibrosis (CF) and can cause severe infections in individuals with compromised immune systems.Colmer-Hamood et al. (2016); Murray et al. (2022) During infection, *P. aeruginosa* forms biofilms that render it highly resistant to antibiotics.Flemming et al. (2016) In these biofilms, bacteria are encased within an extracellular polymeric substance (EPS) matrix composed of polymeric molecules, including, DNA, and proteins.Yan and Bassler (2019); Flemming and Wingender (2010); Böhning et al. (2025) A notable feature of *P. aeruginosa* biofilms is the increased production of filamentous viruses, known as Pf viruses, during biofilm formation.Burgener et al. (2019); Manos et al. (2008); Whiteley et al. (2001) Secor *et al*. demonstrated that these rod-like particles maintain a symbiotic relationship with *P. aeruginosa* in biofilms.Secor et al. (2015); Böhning et al. (2023) Pf4 viruses, with diameters of approximately 6.5 nm and lengths up to 3.8 *µ*m as measured via electron micrographs,Tarafder et al. (2020) can form lyotropic liquid crystals. In the presence of a non-adsorbing polymer, such as alginate, a major component of the EPS secreted by the bacteria themselves,Nivens et al. (2001) the depletion effect facilitates a phase separation into a phase rich in virus particles coexisting with a phase poor in virus particles. Moreover, in the dense phase the virus particles are aligned along a common director forming a nematic (N) liquid crystalline phase, whereas in the poor phase they are randomly oriented forming an isotropic (I) phase.

Such liquid crystalline phase behaviour displayed by mixtures of virus particles and polymer is well-known for certain *model* viruses such as *fd* virus. Dogic and Fraden (2001); Dogic et al. (2004) Its behaviour can largely be understood by entropic arguments commonly used in the field of soft condensed matter: as a function of concentration, particles spontaneously separate into an isotropic and a nematic phase - upon alignment the orientational entropy is reduced but the particles gain translational entropy, a phenomenon first described for hard rod-shaped particles in a seminal paper by Onsager. Onsager (1933, 1949) The addition of non-adsorbing polymer leads to a net attraction between the particles. This depletion attraction is again entropic in origin: polymers are depleted from a zone around the particle’s surface due to entropy. When two such ‘depletion zones’ overlap, the polymers outside the overlap volume effectively push the particles together (osmotic pressure) leading to a net attraction between them, Asakura and Oosawa (1954); Vrij (1976) which opens up the coexistence region of the phase diagram. Lekkerkerker and Tuinier (2011) During phase separation, the nematic phase forms liquid crystal droplets called tactoids, Wang and MacLachlan (2018) which have a characteristic spindle shape, as will be explained later.

In *P. aeruginosa* biofilms, a previous study has shown that these tactoids have a tendency to form around the bacteria, encapsulating the bacterial cells. Tarafder et al. (2020)

During antibiotic treatment, this encapsulation acts as a protective layer, which first was attributed to the high negative charge from large amounts of phage DNA within the nematic droplets.Secor et al. (2015) However, later experiments using ghost filaments (viruses without any genetic material) also showed an increase in antibiotic tolerance. In the presence of just one of the components (either Pf4 or the polymer sodium alginate), the cells are not more tolerant to antibiotics, suggesting that virus formation of tactoids is necessary for this protection.Tarafder et al. (2020) Recently, a study has further shown, using fluorescently tagged antibiotics, that encapsulation of bacteria by phage tactoids is directly correlated to decreased antibiotic uptake into the bacteria suggesting that the Pf4 tactoids are acting as a diffusion barrier. Böhning et al. (2023) Despite this important role of phage tactoids in mediating antibiotic tolerance of pathogenic bacteria and insights into the mechanisms underlying this tolerance, the physical properties driving phage tactoid association with bacterial cells remain unclear.

The formation of tactoids by viruses is well known for *fd*(Dogic and Fraden 2001; Dogic et al. 2004) and tobacco mosaic viruses. Bawden et al. (1936); Urakami et al. (1999) The shape and director field of a tactoid depend on the competition between the elastic properties of the nematic phase, an effective interfacial free energy between the rod surface and the surrounding isotropic or nematic phase, and the anchoring strength at the isotropic-nematic interface. Prinsen et al. (2003); Prinsen and Van Der Schoot (2004) In the nematic phase, perturbations in the director come with an elastic cost, which depends on the type of deformation and is characterized by three elastic parameters. The surface between the isotropic and nematic phase is characterized by an effective interfacial free energy, similar to an interface between ordinary liquids. Prinsen et al. (2003); Prinsen and Van Der Schoot (2004) Lastly, anchoring refers to the preferred orientation of the rod-like particles at a surface, where the anchoring strength is the energetic cost of misalignment from that preferred orientation. It can be categorized as strong or weak anchoring, relative to the elastic energies, De et al. (1993) and is experimentally related to surface chemistry and topography. Guo et al. (2019) The values of the elasticity, surface, and anchoring terms, are functions of the length, aspect ratio, and stiffness of the elongated particles. De et al. (1993); Odijk (1986) In addition, they will depend on the polymer concentration,Alexe-Ionescu et al. (2008); Hakemi (1999); Hamaneh and Taylor (2008) and the ionic strength of the dispersion, Barbero and Evangelista (2005) further complicated by using a polyelectrolyte such as alginate, which is as already mentioned biologically motivated. Lee and Mooney (2012)

**Fig. 1:**
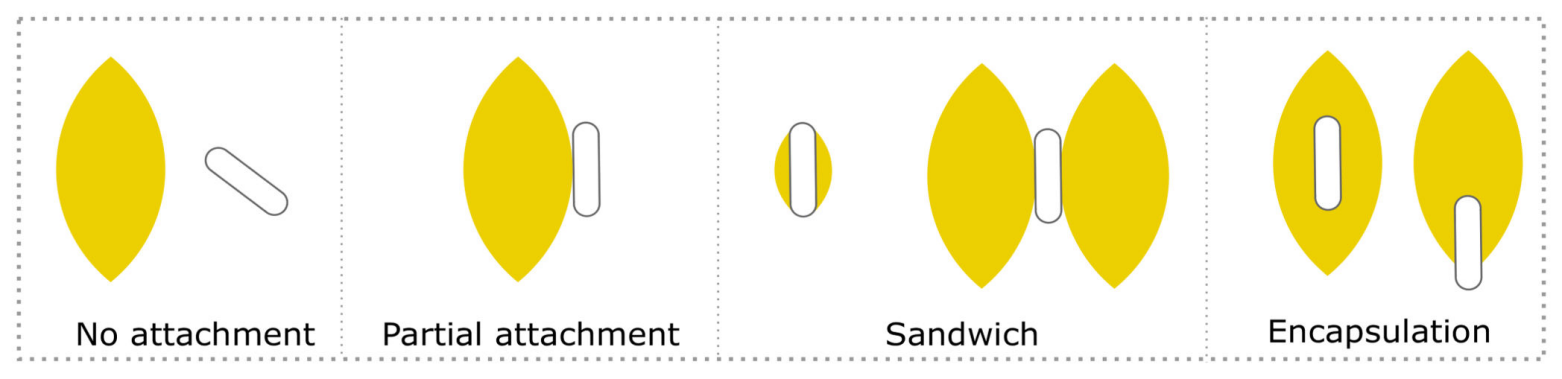
Schematic of assigned configurations, experimentally observed, of the rod (in white) and the nematic droplet, or tactoid (in yellow)

In this work, we introduce a third body to the dispersion of virus particles and polymer. We have used two different types of bacteria (Gram-positive and Gram-negative) as well as synthetic rod-shaped particles. From this point onwards we will refer to this third body either as bacteria or as rods. We will take care to refer to the rod-like virus particles as virus particles or viruses. Of course, the introduction of a third particle further complicates the physics and brings with it an effective interfacial free energy between the rod and the isotropic or nematic phase, as well as an anchoring term between rod and nematic phase. In these experiments, we identified four different states (Fig. 1) when studying the mixture of virus, polymer, and rod: (i) no attachment, where the rods and tactoids do not interact and remain separated; (ii) partial attachment, if the outside of one tactoid attaches to one of the long sides of the rod; (iii) a ‘sandwich’ state, when the outside of two tactoids attach to the same rod; and (iv) encapsulation, if the majority of the rod is inside a tactoid. The focus of the paper will be on configurations where some interaction occurs, i.e. we will not focus on the no attachment state.

We aim to experimentally describe the different configurations of phage tactoid association with bacterial cells using model bacterial organisms and synthetic rods to determine the underlying physical principles driving phage tactoid association with rod-shaped particles. To this end, we have performed a range of experiments, on different bacterial rods as well as on different colloidal model rods. State (iv), encapsulation, is the most interesting state (Tarafder et al. 2020) and we examine this state within a simplified, two-dimensional Oseen-Frank framework, where only the elastic energy is taken into account (2). We then introduce the experimental system (Sect. 3), followed by a presentation of the experimental results (Sect. 4). The experimental results section is split into bacterial rods (Sect. 4.1) and colloidal rods (Sect. 4.2), all mixed with a fixed concentration of Pf4 virus and sodium alginate polymer. This is further divided into model organisms of the two main domains of bacteria membranes, *E. coli* (Gram-negative), and *B. subtilis* (Gram-positive), see Sect. 4.1. In addition, we study two synthetic colloidal rods, silica and SU8 rods, which allows us to explore certain surface effects, such as surface charge (zeta potential), wettability, and surface roughness. Using synthetic rods furthermore facilitates to study the effect of size and aspect ratio of the rods (Sect. 4.2). The experimental results are subsequently discussed in the light of the numerical results (Sect. 4.3).

## Computational methods

As mentioned above, the encapsulation state (iv) is biologically the most interesting state. In order to have some understanding of the elastic terms at play we work with the following simplified model: a 2D tactoid with a fixed circular or spindle-like shape described by the intersection of two congruent circles and centred at the opposite shift value in the x-axis (see supl. fig. 1). The radius and shift in the x-axis (*s*_*x*_) is determined based on the aspect ratio and desired area. Given this fixed configuration we subsequently follow the work done in Lewis et al. (2014) for the nematic phase in rectangular confinement.

For this 2D system, the director can be expressed as a unit vector in the xy-plane by

(1) $$\text{n}=\{\begin{array}{l} \text{cos}(\theta (x,\;y)),\\ \sin(\theta (x,\;y)). \end{array}$$

The Oseen-Frank energy can then be written in terms of *θ* as

(2) $$E[\theta ]=\frac{K_{3}}{2}{\int \int }_{\Omega }|\nabla \theta {|}^{2}-\delta {\left(\theta_{y}\;\text{cos}\;\theta -\theta_{x}\;\text{sin}\;\theta \right)}^{2}\;d\Omega ,$$

where *δ* = 1 − *K*_1_*/K*_3_, and *K*_1_ and *K*_3_ are the elastic constants for splay and bend. We have previously shown in experiments on the semiflexible elongated virus fd that the ratio *K*_1_*/K*_3_ lies around 1. Dammone et al. (2012) As pf4 virus has a similar nature,Nivens et al. (2001) we will assume the single-constant approximation for the Oseen-Frank energy. This results in the expression:

(3) $$\nabla^{2}\theta =0.$$

To solve this, we assume that the director field has two point defects at the tips of the tactoid, in line with experimental images, using the following boundary conditions:

(4) $$\theta =\{\begin{array}{ll} \text{arctan}\left(\frac{y}{x+s_{x}}\right)+\frac{\pi }{2} & \;\text{for}\;\text{right}\;\text{side}\;\\ \text{arctan}\left(\frac{y}{x-s_{x}}\right)+\frac{\pi }{2} & \;\text{for}\;\text{left}\;\text{side}\; \end{array}$$

When using strong anchoring (Dirichlet boundary conditions) *θ* is discontinuous at each tip of the tactoid, creating a point defect with infinite energy. The simplest way to deal with these defects is to create a regularized domain (supl. fig. 2), in which a circle of radius *ϵ*, of the order of the nematic correlation length, is eliminated from the energy calculation. In our case, we use *ϵ* = 0.005 based on literature values (Lewis et al. 2014) for a related system. We solve the equation for the entire tactoid and then obtain the regularized energy for the regularized domain.

To complete the model, we add a spherocylindrical rod (similar shape to the bacteria) to the tactoid, positioned at the long axis of the tactoid. We assume strong anchoring at the surface of the rod, using the following Dirichlet boundary conditions:

(5) $$\theta =\{\begin{array}{ll} \text{arctan}\left(\frac{y-l_{rod}/2-cy}{x}+\frac{\pi }{2}\right) & \text{top},\\ \text{arctan}\left(\frac{y+l_{rod}/2-cy}{x}+\frac{\pi }{2}\right) & \text{bottom},\\ \frac{\pi }{2} & \text{sides}. \end{array}$$

The addition of a rod with strong anchoring adds two further defects at the tip of the rod; the same idea of regularization is applied here (supl. fig. 2).

Note that this two-dimensional Oseen-Frank model does not capture the discrete particle nature of the nematic phase, the potential flexibility of individual viruses, or the specific microscopic interactions at the virus-rod interface. It is used solely to get additional insights into the elastic deformation of the nematic director field in the tactoid and around the impurity.

## Experimental methods

**Fig. 2:**
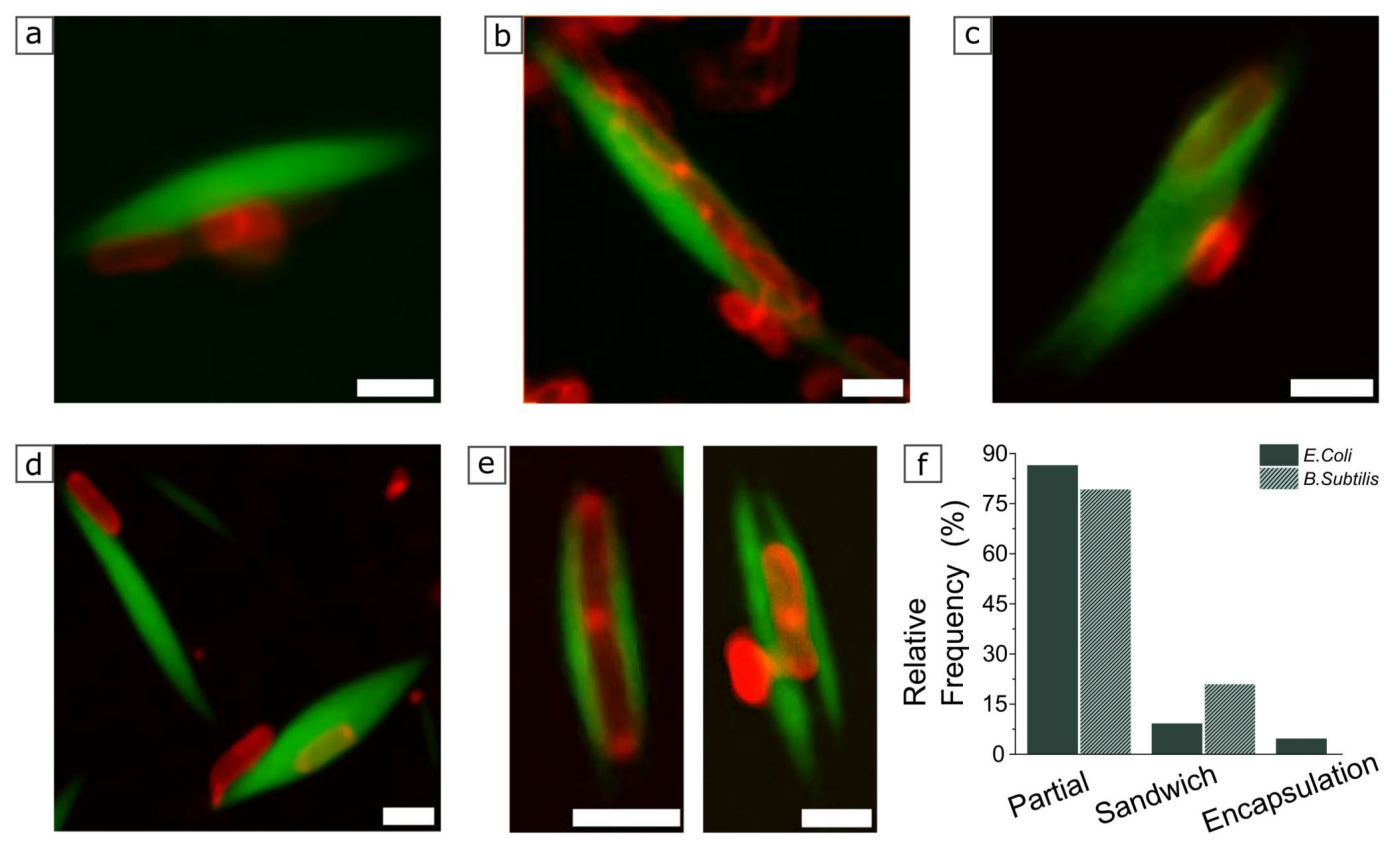
Configurations obtained with bacteria as rods. Confocal microscopy images of bacteria visualised by FM4-64 staining, in red, (**a, b, c**) *E. coli* and (**d, e**) *B. subtilis*, and their different configurations with Pf4 tactoids, in green. (**a, d**) Partial attachment, (**b, e**) sandwich state and (c) encapsulation; (**f**) Histogram of the observed states. The scale bar represents 2 *µ*m.

**Fig. 3:**
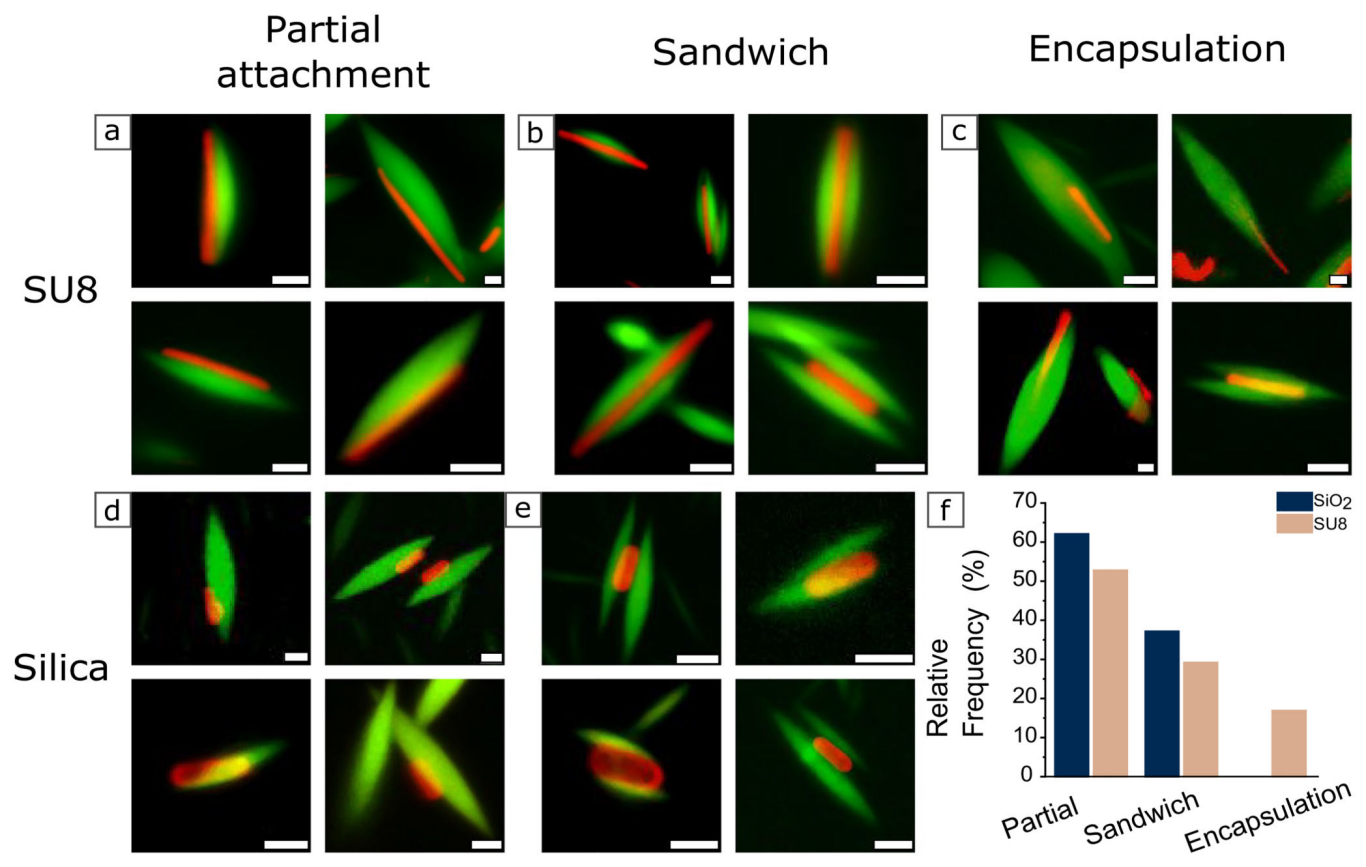
Configurations obtained for the synthetic rods. Confocal microscope images of (**a, b, c**) SU8 and (**d, e**) silica rods, in red, interacting with Pf4 tactoids, in green. (**a, d**) Partial attachment, (**b, e**) sandwich state, and (**c**) encapsulation; (**f**) histogram of the observed configurations. The scale bar represents 2 *µ*m

Experimental details are provided in the Supplementary Material for the chemicals used, propagation of Pf4 virus, *B. subtilis* and *E. coli* bacteria, and synthesis of colloidal rods, silica and SU8. Briefly, after growth/synthesis, the rods were mixed with fluorescently labelled virus at a final concentration of 1 mg/ml and with the sodium alginate at a concentration ranging from 2 to 5 mg/ml. The size of the tactoids depends strongly on the concentration of the biopolymer and on the time after preparation. The phase behaviour of the Pf4–alginate system has been characterised in detail in the first author’s PhD thesis (de Oliveira and Silva 2022) [manuscript in preparation]. There, a broad range of Pf4 (1–16 mg/mL) and sodium alginate (0.05–15 mg/mL) concentrations were explored by confocal microscopy, yielding an experimental phase diagram in the Pf4–alginate plane. Three regimes were identified: (i) a homogeneous isotropic phase at low Pf4 and/or low alginate concentrations, (ii) a biphasic region where nematic Pf4-rich droplets (tactoids) coexist with an isotropic background, and (iii) at higher Pf4 but lower alginate concentrations, a biphasic network regime where polydisperse rods form percolating nematic strands. The Pf4 (1 mg/mL) and alginate (2–5 mg/mL) concentrations used in the present tactoid–rod experiments lie well within the biphasic tactoid regime of this phase diagram. In this window, the thesis work showed that lowering the alginate concentration slows tactoid nucleation and yields larger, more slowly coarsening tactoids, whereas higher alginate concentrations lead to rapid nucleation of many smaller tactoids or network structures. These trends are fully consistent with our present observations (Figs. 2 and 3) and with a depletion-driven phase-separation mechanism. Because of this, different concentrations and different times were used to allow multiple sizes of tactoids to be obtained. Notice that using alginate allows us to closely mimic the physicochemical conditions of the native biofilm environment, which is the ultimate context for the biological phenomenon we are studying. After adding all the components in a vial, the mixture was vortexed for 5 min and then the sample was placed in the square capillary and sealed with epoxy glue, fixed to a glass slide. Then, the system was imaged using a Zeiss LSM Exciter 5 (Confocal Laser Scanning Microscope) with a 63x Zeiss Plan Apo Chromat objective. This allowed the visualization of the interactions between the rods and tactoids (Videos 1 and 2).

## Results and discussion

In this section, we will first present the experimental results for the bacteria, *E. coli* and *B. subtilis* (Sect. 4.1), followed by the experimental results for the synthetic colloidal rods, silica, and SU8 (Sect. 4.2). We will then present the results from the numerical calculations (Sect. 4.3), and compare and contrast our findings.

### Experiments with bacteria

There are two model organisms for the two major forms of bacterial cell envelopes: *E. coli* for Gram-negative (containing two membranes and lipopolysaccharide) and *B. subtilis* for Gram-positive bacteria (containing a single membrane covered by a thick peptidoglycan layer). To assess if the type of cell species plays a role, we have studied both bacteria types. Figure 2 presents typical confocal microscopy images for each of the identified configurations as described in Sect. 1 (Fig. 1). As can be seen from the distribution plot, Fig. 2f, for both types of bacteria, partial attachment is most commonly observed, followed by the sandwich state. Encapsulation is only observed for *E. coli*. The adherence of bacteria to the Pf4 is expected considering the depletion interaction: the free alginate polymer will push the virus particles against any surface, such as the surface of a bacterium. This was also observed by Tarafder et al. (2020), where electron microscopy images showed that the virus was aligned to the outer membrane of the bacteria.

The major difference between the two types of bacteria is the absence of the encapsulated state for *B. subtilis*, the model Gram-positive bacterium. Its cell surface does not have an outer membrane containing lipopolysaccharide (LPS) (the molecule that covers 75 % of the area of the outermost part of the bacterial membrane in Gram-negative bacteria) (Le Brun et al. 2014) and instead ends in peptidoglycan. The surface of *B. subtilis* has a different molecular assembly and chemical composition than *E. coli*, which probably affects the anchoring of the virus with the bacteria, directly but also possibly indirectly due to the difference in interaction of alginate with LPS or peptidoglycan. Indeed, Tarafder et al. (2020) showed that encapsulation did occur for *P. aeruginosa*, which is a Gram-negative bacterium as *E. coli*, and is the natural host for Pf4 viruses.

There are, however, some apparent differences with the work on *P. aeruginosa*. A distribution of states was not presented in Tarafder et al. (2020), but it is clear that a high occurrence of encapsulation was observed. One possible explanation for this difference is the lipopolysaccharide (LPS): the composition of this molecule depends on multiple factors, including the bacteria species.Raetz and Whitfield (2002); Bertani and Ruiz (2018) This may lead to a change in surface morphology/interactions, in turn affecting the anchoring orientation and strength.Selinger (2016) Another possible explanation relates to differences in experimental setup, which will be discussed further down (supl. fig. 3).

The results for bacteria demonstrate that surfaces interactions play an important role, but other factors such as geometry may play a role too, which brings us to the next section, where the system’s configurations around inert colloidal rods of different dimensions and surface properties are examined.

### Experiments with synthetic rods

We synthesized rods made of silica and SU8 with a spherocylindrical shape and similar size to the bacteria (Table 1) to address surface and geometry properties. Both colloidal rods are negatively charged, but SU8 is hydrophobic while silica is highly hydrophilic.Kumar and Sharma (2015)

For both rods we observed: no attachment, partial attachment, and the sandwich state. Encapsulation was only observed for SU8 rods (Fig. 3 a-e). The partial attachment and sandwich states were seen in similar frequencies for both rods, with partial attachment being the most common configuration (Fig. 3f and videos 1 and 2). This is similar to the results for the bacteria. Interestingly, silica rods are more similar in wettability and *ζ* potential to *P. aeruginosa* than the SU8 rods are, but encapsulation was observed for SU8, suggesting that multiple surface properties are at play.

We also note that the rods were typically located towards the tip of the tactoids, especially for encapsulation. Furthermore, for the sandwich state, tactoids are seen to ‘wet’ the rods, i.e. the tactoid seems to spread on the colloidal surface, especially when the tactoid and rod are of similar length. This means that they lose the curvature in half of the droplet, maintaining a straight interface in contact with the side of the rod. This was observed more frequently for the SU8 rods, which are generally longer than the silica rods, but equally have different surface properties.

**Fig. 4:**
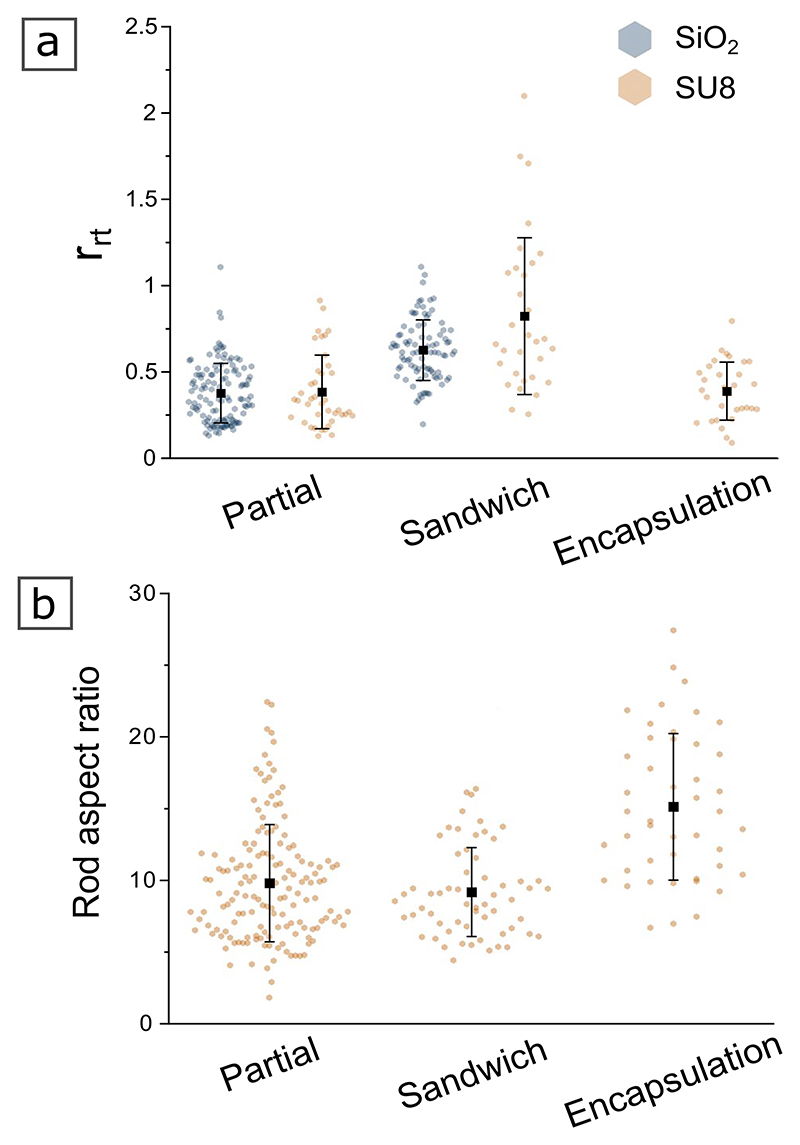
Role of rod-tactoid and rod geometry. Each dot is an experimental sample of a tactoid-rod configuration. x-axis: the 3 type of tactoid-rod configurations. Each column scatter distribution represents the experimental configurations for (**a**) *r*_rt_ = *l*_rod_*/l*_tactoid_) (average ratios ± standard deviation in black) and (**b**) the aspect ratio of the SU8 rods *ar* = *l*_rod_*/d*_rod_

Since a number of states is possible for each type of rod (both synthetic and bacterial), with a range of surface properties, we now focus on geometrical parameters, which is most easily done for the synthetic rods, since they are easier to image and cluster less. From the confocal images we obtain the ratio between the length of the rod and the length of the tactoid: *r* = *l*_rod_*/l*_tactoid_. A beeswarm plot of each configuration is presented in Fig. 4a. Silica and SU8 rods show similar results (average value and standard deviation) for partial attachment and the sandwich state, but the sandwich state is more frequently observed for larger *r*. This suggests that rods tend to get sandwiched when their size is comparable to the length of the tactoid. The data for encapsulation (SU8 only) are again similar in average and standard deviation to those for partial attachment.

Figure 4b recasts the results for SU8 rods as a function of the rod’s aspect ratio *ar* = *l*_rod_*/d*_rod_, with *l* and *d* the length and thickness of the rod. Here, it becomes apparent that long, thin rods are more prone to encapsulation. This may be due to the role defects play in encapsulating rods, which we will examine further in the next section. This observation suggests that long, thin *silica* rods may be more easily encapsulated. To this end, silica rods with a higher aspect ratio were synthesized, but a preliminary study shows that no cases of encapsulation are observed. At the same time one should keep in mind that the aspect ratio of the SU8 rods is much larger than of the bacteria (Table 1). This again highlights that a combination of surface properties and geometry determines the occurrence of the different states, which to some extent can be more easily explored numerically.

### Numerical results

Here, we evaluate how adding a rod to the tactoid impacts the director fields as well as the regularized elastic energy, as a function of the rod’s relative size and position, which is related to the experiment.

**Fig. 5:**
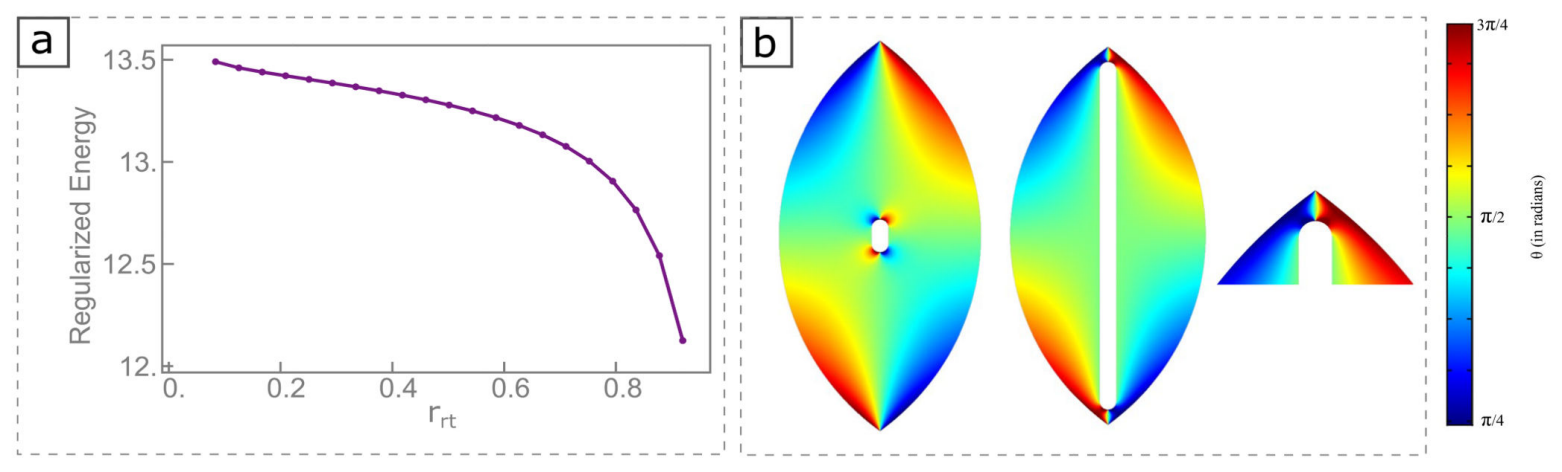
(**a**) Regularized energy of the system as a function of the normalized rod length (rod length divided by tactoid length). (**b**) Visualizations of the director field for a tactoid with rods of varying lengths positioned at the center. The color scale represents the orientation of the tactoid’s director field

First, we examine how the energy changes with rod length while keeping the diameter constant. The regularized energy is calculated as a function of *r*_rt_ = *l*_rod_*/l*_tactoid_. When a rod is introduced, the energy increases (from 8. to 13.5) due to the additional elastic cost of aligning the director field around the rod and the creation of a point defect at the tip of each rod. As the rod length increases, the energy decreases (Fig. 5a). The energy decrease is relatively small (from 13.5 to just above 12), but shows that it is energetically more favourable to capture thin long rods as opposed to short fat ones. This is likely due to the proximity of defects at the tip of the tactoid and at the tip of the rod, see Fig. 5b. The defect at the tactoid tip has a positive charge, while the defect at the rod tip is negative, leading to an attractive interaction that reduces the director field distortion and lowers the elastic energy. As a result, energy minimization occurs when the rod and tactoid lengths are comparable.

**Fig. 6:**
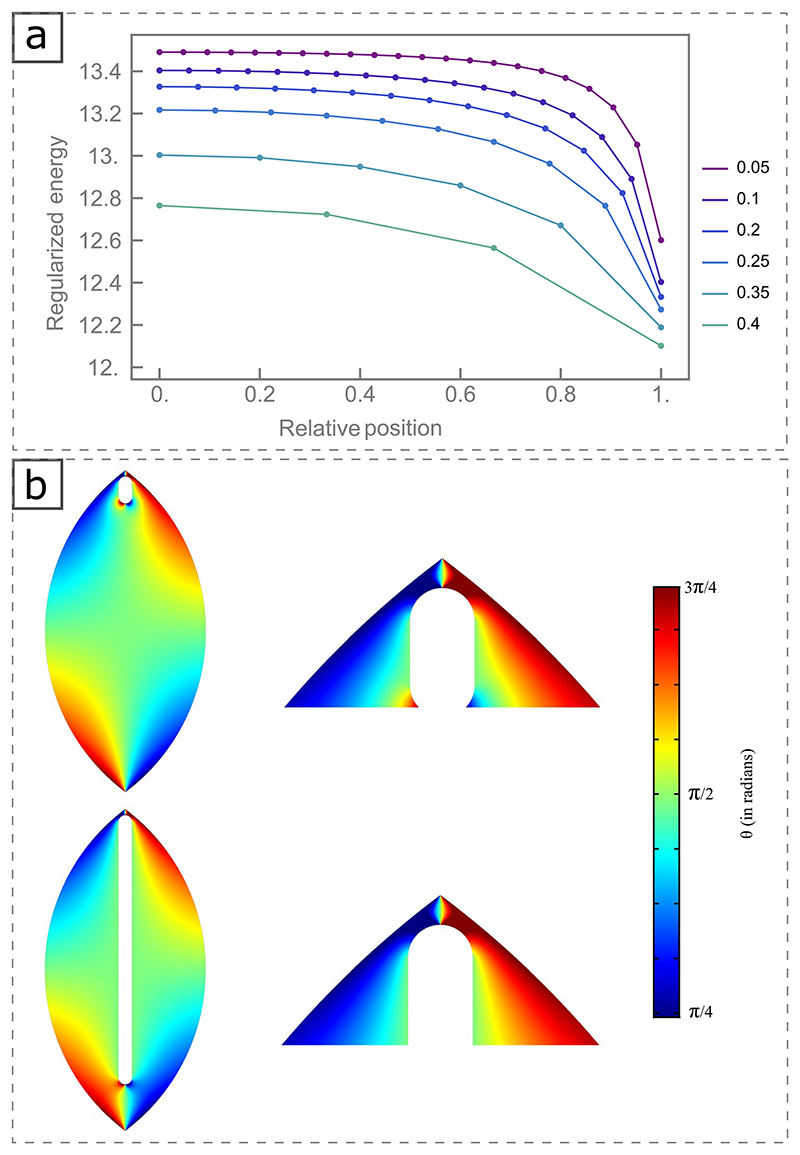
(**a**) Regularized energy of the system by relative position between rod and tactoid for different normalized rod lengths (from 0.05 to 0.4). The minimum position (0) corresponds to the tactoid being in the center. The maximum position (1) is the farthest point from the tactoid center where the rod still remains inside without touching the boundary. (**b**) Images of the director field for tactoids with rods of different sizes at different positions, and magnification for the position closest to the tip. The color scale represents the orientation of the tactoid’s director field

Next, we investigate the effect of the position of the rod along the long axis of the tactoid. To facilitate comparison across rods of different lengths, we use the position of the rod divided by its maximum position, which is the point where the rod remains inside the tactoid without touching the tactoid boundary) (Fig. 6), i.e. a relative position of 1 corresponds to the rod being at the tactoid tip, while 0 corresponds to it being at the center. The results show that moving the rod closer to the tactoid tip reduces the energy (Fig. 6a). This is in line with experimental observations, where most encapsulated states exhibit rods positioned near the tactoid tips (Fig. 3c); it furthermore suggests that the proximity of defects reduces the energy. Note that longer rods have lower energies, but the energy decreases more sharply for thicker rods, where director field distortions are greater, when moved to the tip.

Finally, we also investigated the effect of changing the rod diameter, for fixed rod length. Results are as expected: thinner rods have lower energies, and the energy decreases when the rod is moved closer to the tip of the tactoid (supl. fig. 4).

These findings highlight the role of defect interactions in determining the relative energies of tactoid-rod configurations. The experimental preference for rods near tactoid tips can be understood within this continuum framework. To fully interpret the experimental observations would require a 3D calculation, with free boundaries, or 3D particle simulation, which falls outside the scope of the present work.

## Conclusions and outlook

Phage tactoids have been shown to increase antibiotic tolerance of the pathogenic bacteria, *P. aeruginosa*, by associating to the bacteria and forming a diffusion barrier, reducing the uptake of antibiotic into the cell.Böhning et al. (2023) To understand the physical properties of the tactoid and bacteria that drive this association, we studied the possible configurations of tactoids in the presence of different rods. Four configurations were obtained: no attachment, partial attachment, sandwich state and encapsulation. The three first states were observed for all the rods tested, while encapsulation was only seen for *E. coli* and SU8 rods. We determined a geometrical relationship between configuration and the size of the rod and of the tactoid - partial attachment and encapsulation are seen for rods smaller than the tactoid, with the latter also requiring rods with long aspect ratio (thinner and longer), and the sandwich state occurs when the tactoid length is similar to the rod length. Encapsulation was only observed for Gram-negative bacteria and SU8 rods, clearly pointing to the importance of surface properties.

Calculations within the one constant approximation on model 2D configurations highlighted the interplay between director fields and defects. It showed that the elastic energy was minimized for longer encapsulated rods, and that the elastic energy decreased as the rod approached the tactoid tip, since defects of opposite charge are brought together. Experimentally, rods in the encapsulation configuration were often located near the tactoid tip, consistent with this finding. Moreover, the thin long SU8 rods were more likely to be encapsulated, in line with the computational observations. A full 3D calculation with free boundaries falls outside the scope of the current work, but we hope that this challenge may be picked up in simulation or theoretical studies: it is a rich and complex *fundamental* problem with *real-world* implications. Of course further questions emerge around the use of continuum vs discrete models, with rods that are semi-flexible, and where (surface) charges are present everywhere. We also note that our physical approach does not account for potential specific biomolecular interactions between phages and bacteria, such as receptor-ligand binding, which may further modulate adhesion and encapsulation in the native biological system. Valentini et al. (2018)

Finally, encapsulation was typically less frequently observed than in the original work with *P. aeruginosa*. This may be connected to the different setups: in the original work, the system was confined between an agar pad and a cover slide constituting a quasi-2D setup. This is in contrast to the capillaries used here, where the bacteria are in bulk 3D. To check whether this confinement could affect the configurations obtained, we repeated our experiments with all types of rods using a similar setup as in Tarafder et al. (2020) (supl. fig. 3). Importantly, we found that the tactoid shapes were different than in 3D, and indeed in this more confined setup we did see that *B. subtilis* and silica rods became encapsulated too. The observation that encapsulation occurs more frequently in quasi-2D confined systems raises questions about the role of elastic anisotropy. In our numerical calculations, we adopted the one-constant approximation, which assumes equal energetic cost for splay and bend deformations. Under quasi-2D confinement, however, bend deformations in the direction perpendicular to the confinement plane are restricted by the confining walls. This may alter the balance between elastic energies, potentially favouring encapsulation.

We have analysed this problem within a continuum 2D model. Next steps could be the extension to calculations in 3D or quasi-2D, and considering the isotropic-nematic interface as a free boundary. Moreover, given that the length of the virus is comparable to the length of the rods, it is likely that continuum models start breaking down and although the experimentally observed tactoid shapes can, to some extent, be described by the continuum models, it is known from related experiments on strongly confined colloidal crystals in quasi 2D chambers, that finite size effects may lead to novel equilibrium states or long-living metastable states, not captured by the theoretical models. Gârlea et al. (2019, 2016); Lewis et al. (2014) This is especially relevant when looking at the defects which have to be artificially included in the theoretical models, but will be of a different nature in the experiments given the size of the viruses as well as their flexibility.

## Supplementary Material

**Supplementary Information** The online version contains supplementary material available at https://doi.org/10.1007/s00249-026-01844-5.

## Figures and Tables

**Table 1:** Size and zeta (*ζ*) potential (related to surface charge) of synthetic rods and bacteria used in the work, and of *P. aeruginosa* for comparison

Rod	Length (μm)	Diameter (μm)	*ζ* (mV)
Thick	2.43 ± 0.26	0.98 ± 0.06	−13.9
Thin	1.67 ± 0.20	0.41 ± 0.06	−12.1
SU8	3.70 ± 1.60	0.52 ± 0.17	−1.4
*E. coli*	1.5 to 1.9 Bolster et al.(2009)	0.6 to 1 Bolster et al. (2009)	−22 to −48 Bolster et al. (2009); Arakha et al. (2015); Zhang et al. (2022)
*B. subtilis*	2 to 6 Errington and van der Aa (2020)	<1 Errington and van der Aa (2020)	−17 to −34 Arakha et al. (2015); Zhang et al. (2022)
*P. aeruginosa*	1 to 5 Diggle and Whiteley (2020)	0.5 to 1 Diggle and Whiteley (2020)	−7 to −12 Gottenbos et al. (1999); Liu et al. (2007)

## Data Availability

No datasets were generated or analysed during the current study.
